# Neurofeedback and the Aging Brain: A Systematic Review of Training Protocols for Dementia and Mild Cognitive Impairment

**DOI:** 10.3389/fnagi.2021.682683

**Published:** 2021-06-09

**Authors:** Lucas R. Trambaiolli, Raymundo Cassani, David M. A. Mehler, Tiago H. Falk

**Affiliations:** ^1^Basic Neuroscience Division, McLean Hospital - Harvard Medical School, Boston, MA, United States; ^2^Institut National de la Recherche Scientifique - Energy, Materials, and Telecommunications Centre (INRS-EMT), University of Québec, Montréal, QC, Canada; ^3^Institute for Translational Psychiatry, University of Münster, Münster, Germany

**Keywords:** neurofeedback, dementia, Alzheimer's disease, mild cognitive impairment, electroencephalography, functional magnetic resonance imaging

## Abstract

Dementia describes a set of symptoms that occur in neurodegenerative disorders and that is characterized by gradual loss of cognitive and behavioral functions. Recently, non-invasive neurofeedback training has been explored as a potential complementary treatment for patients suffering from dementia or mild cognitive impairment. Here we systematically reviewed studies that explored neurofeedback training protocols based on electroencephalography or functional magnetic resonance imaging for these groups of patients. From a total of 1,912 screened studies, 10 were included in our final sample (*N* = 208 independent participants in experimental and *N* = 81 in the control groups completing the primary endpoint). We compared the clinical efficacy across studies, and evaluated their experimental designs and reporting quality. In most studies, patients showed improved scores in different cognitive tests. However, data from randomized controlled trials remains scarce, and clinical evidence based on standardized metrics is still inconclusive. In light of recent meta-research developments in the neurofeedback field and beyond, quality and reporting practices of individual studies are reviewed. We conclude with recommendations on best practices for future studies that investigate the effects of neurofeedback training in dementia and cognitive impairment.

## 1. Introduction

Dementia describes a set of symptoms that occur in neurodegenerative disorders caused by damage and death of neurons. These symptoms include gradual loss of cognitive, affective and behavioral functions, leading to increased impairment in activities of daily living (Livingston et al., 2017). Dementia mostly affects people over the age of 65 years and its incidence rows exponentially with age (Prince et al., 2015). For instance, while the prevalence of dementia in the age range of 70–90 Hz years is between 15 and 20% of the population, it is higher than 40% for 90+ years old (Plassman et al., 2007; Corrada et al., 2008, 2010). Further, given expected demographic developments, dementia presents a major concern for many societies. While elderly people currently account for about 12% of the world population, this proportion is expected to grow to 21% by 2050 (Wasay et al., 2016), with projections pointing to more than 130 million people with dementia by then (Prince et al., 2015).

Symptoms in dementia are complex and affect different domains, including cognitive (e.g., memory loss, mental confusion, language impairment), behavioral (e.g., irritability, personality changes), and psychological functions (e.g., anxiety, depression, hallucinations), leading to increasing levels of dependency as the disease progresses (World Health Organization, 2012; Prince et al., 2015). Among the biological causes for dementia, the most common in order of frequency are: Alzheimer's Disease (AD), which accounts to nearly 70% of the dementia cases, vascular dementia (VD), Lewy body dementia (LBD), and frontotemporal dementia (FTD) (World Health Organization, 2012). Another neurodegenerative disorder that leads to dementia in patients is Parkinson's disease (PD) (Gratwicke et al., 2015). Neurofeedback studies have been successfully conducted with PD patients to treat motor symptoms (Linden and Turner, 2016; Subramanian et al., 2016). To our knowledge, however, and in contrast to neurofeedback based rehabilitation in stroke (Wang et al., 2018), no neurofeedback study to-date has targeted cognitive symptoms in PD patients. Lastly, mild cognitive impairment (MCI) constitutes another disorder that features cognitive impairment. These deficits exceed those that are commonly observed in normal aging, but are less severe compared to mild forms of dementia. Noteworthy, MCI is associated with a higher risk to develop dementia (Petersen, 2004, 2011; Mariani et al., 2007; Plassman et al., 2007). For instance, longitudinal evaluations have shown that more than 25% of patients with MCI develop AD in subsequent years, which is a much higher conversion rate compared to a healthy aging population (Boyle et al., 2006; Brodaty et al., 2014). On the other hand, a substantial proportion of patients diagnosed with MCI may recover to cognitive levels comparable to their age group (Ganguli et al., 2011; Han et al., 2012; Klekociuk et al., 2016). MCI patients may hence benefit in particular from complex cognitive interventions, such as neurofeedback training.

Current therapies for dementia focus on providing temporary symptom improvement and reducing the rate of cognitive decline, but are minimally effective to slow down disease progression (Koyama et al., 2012). Also, existing drugs are not effective for all types of dementia, nor for different severity levels of the same disease. For example, although cholinesterase inhibitors are effective at reducing symptoms in patients with mild to moderate AD, they show negligible effects in patients presenting with very mild or severe symptoms (Cummings et al., 2002; Cummings, 2004). For other types of dementia, such as VD, there is currently no specific drug available, and patients are usually treated off-label with substances that are used to treat AD patients Erkinjuntti et al. (2004). Moreover, there are no approved efficacious treatments for MCI (Karakaya et al., 2013). Taken together, there is an urgent need to develop effective interventions that can slow disease progression (Hogan et al., 2008). Non-invasive neurofeedback training has been suggested as a potential complementary treatment for dementia. During this intervention, patients actively engage in cognitive tasks and modulate the activity in areas that show correlated activity and that are selected based on a pathophysiological disease model (Kim and Birbaumer, 2014; Sitaram et al., 2017).

### 1.1. Neurofeedback Systems

Neurofeedback protocols aim to train users to achieve self-regulation of specific neural substrates through real-time feedback (Kim and Birbaumer, 2014; Sitaram et al., 2017). This learning process is grounded in operant conditioning (or reinforcement learning) such that desired brain activity is rewarded (Ros et al., 2014). Neurofeedback systems consist of three main components: an imaging modality (e.g., functional magnetic resonance imaging), a series of signal processing steps to extract and filter relevant (i.e., ideally neural) information, and a feedback presentation of this information to the user.

Two different groups of neuroimaging techniques are used for neurofeedback studies: Electrical or magnetic signals (that result from dipole sources of electrical, neural activity) (Min et al., 2010) are the basis for electroencephalography (EEG) (Enriquez-Geppert et al., 2017) and magnetoencephalography (MEG) (Parkkonen, 2015). These neuroimaging techniques possess high temporal resolution (of up to 100 kHz in modern equipment), allowing for high sampling rates and thus frequent updates of presented neurofeedback. Blood oxygenation level (i.e., hemodynamic responses) forms the basis for functional magnetic resonance imaging (fMRI) (Weiskopf, 2012; Paret et al., 2019) and functional near-infrared spectroscopy (fNIRS) (Kohl et al., 2020). These imaging techniques thus provide an indirect measure of neural activity, which results from the metabolism of brain cells (Min et al., 2010). fMRI and fNIRS have lower temporal but higher spatial resolution compared to EEG, allowing for more specific targeting of brain structures.

During real-time data processing, recorded signals are converted into an output of the closed-loop system (Sitaram et al., 2017). Ideally, noise-reduction and feature extraction approaches are used to remove artifacts and convert the original time series into standardized and informative measures of neural activity (Gruzelier, 2014). Processing algorithms used to achieve better data quality vary between imaging techniques and regions of interest and remain an active field for methodological research. For example, EEG-based protocols usually involve self-regulation of frequencies or electrical potentials of specific EEG channels (Enriquez-Geppert et al., 2017). On the other hand, fNIRS- and fMRI-based neurofeedback systems focus on the up- or down-regulation of the hemodynamic signal in specific brain areas. Noteworthy, while fMRI allows recording from and thus targeting subcortical areas directly, the spatial resolution of fNIRS is limited to the cortical surface (Kohl et al., 2020).

Feedback presentation constitutes another relevant component of neurofeedback protocols. The goal of most currently employed paradigms is to provide users with real-time about the targeted neural activity, allowing them to adapt their control strategy to achieve a desired level of proficiency (Curran and Stokes, 2003; Birbaumer et al., 2013). Different perceptual modalities can be stimulated, e.g., via auditory, visual, vibrotactile, electrical or proprioceptive feedback systems (Sitaram et al., 2017). The choice and configuration of the feedback modality should be carefully planned because it can interfere negatively with self-regulation performance and learning curves of participants (McFarland et al., 1998; Birbaumer et al., 2013).

### 1.2. Rationale for Using Neurofeedback in the Treatment of Dementia

Although being a relatively new field, preliminary studies with healthy participants and different clinical populations suggest that neurofeedback training may be effective to improve brain function, treat cognitive as well as affective symptoms and induce brain plasticity (Arns et al., 2017; Sitaram et al., 2017; Thibault et al., 2018). Therefore, neurofeedback training has been suggested as a potential complementary treatment for patients suffering from dementia.

Cognitive decline is a defining feature of dementia. Neurofeedback training combines cognitive training with operant conditioning of the associated neural substrate, e.g., attention or memory recall (Sitaram et al., 2017). For instance, working memory and attention share common neural mechanisms, which can be trained through top-down cognitive strategies (Cicerone et al., 2011; Gazzaley and Nobre, 2012), leading to performance improvements (Karbach and Verhaeghen, 2014). Thus, neurofeedback-based cognitive training may provide an attractive complimentary treatment for patients suffering from different forms of dementia (Jiang et al., 2017). For example, in experiments with healthy elderly participants, EEG-based neurofeedback training led to improved performance in different cognitive domains (Angelakis et al., 2007; Keizer et al., 2010; Lecomte and Juhel, 2011; Becerra et al., 2012; Wang and Hsieh, 2013; Reis et al., 2016; da Paz et al., 2018).

Besides cognitive decline, it is known that more than 70% of patients with dementia experience psychological symptoms, such as anxiety, depression, or apathy (Lyketsos and Lee, 2004; Craig et al., 2005; Steffens et al., 2005). Different neurofeedback protocols were able to target brain areas and networks responsible for emotion processing (Johnston et al., 2010; Linhartová et al., 2019), which opens the possibility of applying this method to treat psychological symptoms in patients suffering from dementia (Kim and Birbaumer, 2014; Arns et al., 2017). For example, randomized controlled trials (RCTs) of fMRI-based neurofeedback training reported improvement of depressive symptoms in patients with major depressive disorder (Young et al., 2017; Mehler et al., 2018) and anxiety symptoms in patients with obsessive-compulsive disorder (Scheinost et al., 2014). For instance, in experiments with older adults, fMRI- and EEG-based neurofeedback training was associated with improved recognition of emotionally valent faces (Rana et al., 2016) and reduced depressive symptoms (Ramirez et al., 2015), respectively.

As previously mentioned, patients with dementia present specific neural signatures that correlate with their symptoms. For example, patients with MCI and AD present altered EEG frequencies (Cassani et al., 2018) and abnormal fMRI functional connectivity at rest (Jacobs et al., 2013; Badhwar et al., 2017). These correlates may provide biomarkers, and their predictive potential as well as validity can be tested by using these as treatment targets (Mehler and Kording, 2018; Micoulaud-Franchi et al., 2019). Similar to brain stimulation protocols, neurofeedback protocols aim to modulate local activity (Linden, 2014). However, since neurofeedback acts as an “endogenous” stimulation, it reduces safety risks or side effects associated with other approaches, such as non-invasive transcranial stimulation or invasive deep brain stimulation. Specifically, it is hypothesized that training participants to regulate biomarkers, it may be possible to induce cognitive improvements, stimulating residual neural plasticity that is maintained to some degree despite dementia (Mirmiran et al., 1996; Prichep, 2007).

To the best of our knowledge, the available literature lacks a systematic review about neurofeedback training to treat dementia. We intend to fill this gap following three aims: First, we summarize and compare current findings reported for neurofeedback studies conducted in patients suffering from dementia or mild cognitive impairment, with special attention to clinical effects reflected in standardized cognitive assessment scales; second, we evaluate the design and reporting quality of these studies according to standardized neurofeedback checklists; and third, we provide guidelines for future research that may help the field progressing.

## 2. Methods

This review followed the Preferred Reporting Items for Systematic Reviews and Meta-Analyses (PRISMA) guidelines (Liberati et al., 2009).

### 2.1. Search Strategy and Study Selection

A survey on peer-reviewed journal articles published in English until October 9th, 2020, was performed for this review. The bibliometric databases Pubmed, Web of Science, IEEE Xplore, Scopus, and ScienceDirect were queried to collect an initial list of papers containing specific search terms in their title or abstract. The following keywords were used in the search:

1. Neurofeedback
2. Neurotherap^*^
3. Dement^*^
4. Alzheimer^*^
5. Lewy^*^
6. Frontotemporal
7. Vascular
8. Cognitive^*^impair^*^

These terms were further combined with logical operators in the following way:

(1 *OR* 2) *AND* (3 *OR* 4 *OR* 5 *OR* 6 *OR* 7 *OR* 8)

Resulting articles were selected or rejected based on the criteria described in Table 1. To assess the eligibility of the selected papers, we first evaluated the titles. If the inclusion or exclusion criteria were not clearly met, the abstract was read as well. Finally, the remaining papers were submitted to full text screening and those found to be misaligned with the eligibility criteria were rejected.

**Table 1:** Eligibility criteria.

**INCLUSION CRITERIA**
1. Studies presenting original results (clinical trials, pilot studies, etc.)
2. Studies including patients with a formal diagnosis of dementia
**EXCLUSION CRITERIA**
1. Studies including samples with other neurological/psychiatric disorders,
or targeting dementia-like symptoms in other disorders
2. Studies exclusively evaluating healthy participants
3. Studies applying biofeedback based only on non-neural signals
4. Studies without voluntary control of brain activity
5. Studies with animal models
6. Review articles, Commentaries, Editorials, and Case Reports (*N* < 5)

### 2.2. Data Extraction and Analysis

To collect relevant information while reading the articles, a data extraction sheet was created including 29 data items which were extracted and grouped into five categories: *population characteristics* (population type, age, gender, education, cerebrospinal fluid (CSF) biomarkers, baseline scores in standardized symptom scales, comorbidity, and current treatment); *study design* (existence of control group, randomization, blinding, evaluation at baseline, at post-training, and at follow-up); *neurofeedback protocol* (imaging method, neurofeedback paradigm, control paradigm, number of sessions, session duration, session description, feedback modality, feedback description, and instruction); *outcomes from standardized cognitive assessment scales* (differences within groups, between groups, and at follow-up); and *other cognitive, behavioral, and neural changes* (differences within groups, between groups, and at follow-up).

We also evaluated each article based on the study design and reporting quality as previously conducted in systematic neurofeedback reviews (Kohl et al., 2020; Trambaiolli et al., 2021). For this, we scored each study according to the checklist for quasi-experimental studies of the Joanna Briggs Institute (JBI) critical appraisal tools (Tufanaru et al., 2017), and the “Consensus on the Reporting and Experimental Design of Neurofeedback studies” (CRED-nf) checklist (Ros et al., 2019). The JBI checklist includes items regarding *clarity of cause and effect, similar participants, similar treatment in compared groups, existence of a control group/condition, multiple measurement points of the outcome, completion of follow-up, similar outcome measurements in compared groups, reliability of outcome measurements*, and *appropriate statistical methods*. On the other hand, the CRED-nf checklist presents a series of 18 essential and eight encouraged items that should be included in neurofeedback design and reports, including *pre-experiment registration, control groups and measures, feedback specifications, outcome description*, and *data storage/publishing*. Deviations from the original criteria in both checklists are detailed in the Supplementary Material.

Data extraction was performed by two independent raters who read the full manuscript and reported independently information to separate extraction sheets. These extraction sheets were later compared to ensure data consistency. In case of disagreement on the extracted/scored items, the final decision was made based on discussions between the two raters.

## 3. Results and Discussion

**Figure 1:**
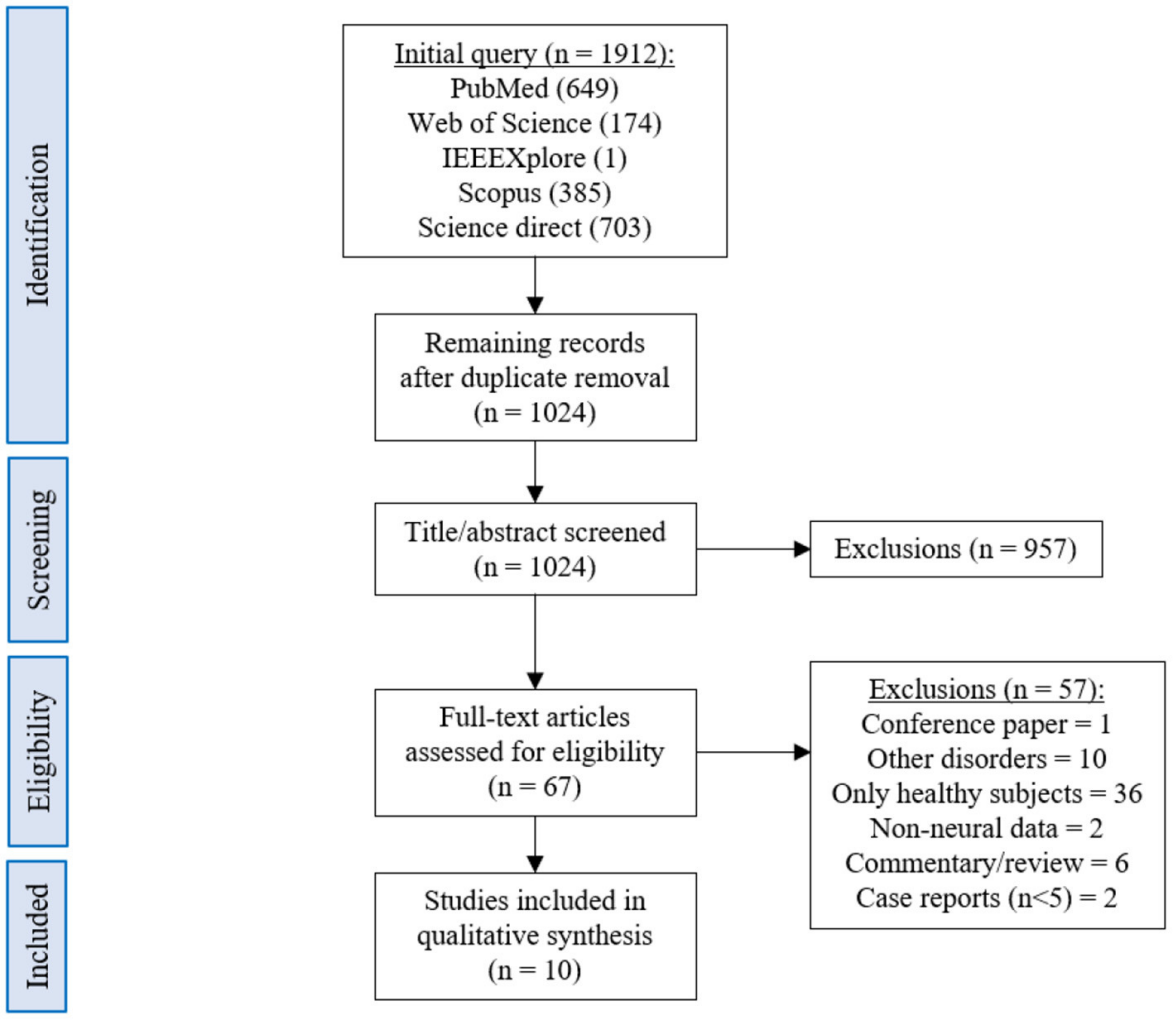
PRISMA flowchart describing the literature screening.

The database queries identified 1,912 studies that matched the search terms (see Figure 1), with 1,024 unique records remaining after duplicates were removed. Through title and abstract screening, 957 studies were rejected because they did not meet the inclusion criteria. After full-text examination, only ten studies were included in the final sample. Of interest, the oldest study in the final sample dates from 2016, and more than half of the studies have been published in the last 2 years, emphasizing the novelty in the use of neurofeedback as a complementary intervention for patients with dementia. Following the first aim of this review, we provide an overview of the selected studies in the next manuscript section, focusing on provided information about enrolled study populations (Table 2), experimental group design (Table 3), neurofeedback protocol (Table 4), and main outcome measures (Tables 5, 6).

**Table 2:** Summary of population characteristics.

**Study**	**Population**	**Age (years)**	**Gender**	**Education (years)**	**CSF biomarkers**	**Symptom scales** ** (baseline avg ± std)**	**Comorbidity**	**Treatment**
**DEMENTIA**								
Surmeli et al. (2016)	NF: 9 AD 11 VD	NF: 68.9 ± 10.6	NF: 9M/11F	NF: 6 (median)	NR	**MMSE:** NF: 18.8 ± 6.4	Psychiatric disorders (anxiety, depression, etc.)	Medicines (*N* = 17)
Luijmes et al. (2016)	NF: 10 poss. AD	NF: 71.5 ± 6.7	NF: 3F/7M	NR	NR	**CAMCOG:** NF: 0.8 ± 0.1	No^*^	Medicines (*N* = 10)
Hohenfeld et al. (2017)	NF1: 10 prom. AD CG1 (real): 16 HC CG2 (sham): 4 HC	NF1: 66.2 ± 8.9 CG1 (real): 63.5 ± 6.7 CG2 (sham): 64.8 ± 9.5	NF1: 8M/2F CG1 (real): 9M/7F CG2 (sham): 3M/1F	NF1: 12 (median) CG1 (real): 13 (median) CG2 (sham): 13 (median)	NF1: Yes CG1 (real): No CG2 (sham): No	MWT-B IQ NF1: 107.8 ± 14.1 CG1 (real): 124.0 ± 9.3 CG2 (sham): 121.5 ± 21.7 MoCA NF1: 24.8 ± 3.2 CG1 (real): 26.8 ± 2.0 CG2 (sham): 26.0 ± 4.2	No^*^	NR
Hohenfeld et al. (2020)	NF: 9 prom. AD CG (real): 12 HC	NF: 64.7 ± 8.3 CG (real): 65.3 ± 6.3	NF: 7M/2F CG (real): 6M/6F	NF: 10 (median) CG (real): 13 (median)	see Hohenfeld et al., 2017	**MWT-B IQ:** NF: 107.3 ± 14.8 CG (real): 126.0 ± 9.4 **MoCA:** NF: 25.1 ± 3.3 CG (real): 26.8 ± 2.2	No^*^	NR
**MCI**								
Mendoza Laiz et al. (2018)	NF1: 22 MCI NF2: 10 MCI	NF1: 66.0 ± 2.2 NF2: 73.1 ± 3.6	NF1: 12M/10F NF2: 2M/8F	NR	NR	MMSE: Between 18 and 23	NR	NR
Jang et al. (2019)	NF: 5 MCI	NF: 66.6 ± 3.5	NF: NR	NF: 9.2 ± 3.6	NR	**MoCA-K:** NF: 19.4 ± 2.1	NR	No
Jirayucharoensak et al. (2019)	NF: 26 HC 32 aMCI CG1 (game): 17 HC 19 aMCI CG2 (CAU): 11 HC 14 aMCI	NF: 71.7 ± 6.5 CG1 (game): 73.9 ± 6.2 CG2 (CAU): 70.9 ± 5.1	NF: 58F CG1 (game): 36F CG2 (CAU): 25F	NF: 9.0 ± 5.7 CG1 (game): 9.4 ± 6.0 CG2 (CAU): 11.4 ± 4.5	NR	MMSE: NF: 27.3 ± 2.1 CG1 (game): 27.5 ± 2.1 CG2 (CAU): 27.9 ± 1.8 MoCA: NF: 22.4 ± 4.3 CG1 (game): 22.8 ± 4.4 CG2 (CAU): 23.6 ± 0.4	No^*^	CAU (type not specified) (*N* = 119)
Lavy et al. (2019)	NF: 11 MCI	NF: 70.0 ± 10.0	NF: 6F/5M	NR	NR	NR	No^*^	NR
Li et al. (2020)	NF: 40 MCI	NF: 54.3 ± 4.9	NF: 20M/20F	NR	NR	NR	No^*^	NR
Marlats et al. (2020)	NF: 22 MCI	NF: 76.1 ± 5.9	NF: 5M/17F	NF: 14.9 ± 2.6	NR	MMSE: NF: 25.4 ± 2.8 MoCa: NF: 23.1 ± 2.5	No^*^	NR

*Symptom scales described as primary outcomes are highlighted in bold*.

* *Studies describing exclusion criteria for other neurological or psychiatric disorders were considered without comorbidities. CAMCOG, Cambridge Cognitive Examination; CAU, Care as Usual; CG, Control Group; MMSE, Mini Mental Status Examination; MoCA, Montreal Cognitive Assessment; MoCA-K, Montreal Cognitive Assessment - Korean version; MWT-B IQ, Multiple Choice Word Test; NF, Neurofeedback; NR, Not Reported*.

**Table 3:** Summary of study design.

**Study**	**Control group**	**Randomization**	**Blinding**	**Evaluation time points**		
				**Baseline**	**Post-training**	**Follow-up (weeks)**
**DEMENTIA**						
Surmeli et al. (2016)	No	No	No	Yes	Yes	No
Luijmes et al. (2016)	No	No	No	Yes (up to 3 months before)	Yes	No
Hohenfeld et al. (2017)	Yes	NR	NR	Yes	Yes	No
Hohenfeld et al. (2020)	Yes	NR	NR	Yes	Yes	No
**MCI**						
Mendoza Laiz et al. (2018)	No	No	No	Yes	Yes	No
Jang et al. (2019)	No	No	No	Yes	Yes	No
Jirayucharoensak et al. (2019)	Yes	Yes	NR	Yes	Yes	No
Lavy et al. (2019)	No	No	No	Yes	Yes	Yes (4)
Li et al. (2020)	No	No	No	Yes	Yes	No
Marlats et al. (2020)	No	No	No	Yes	Yes	Yes (4)

*NR, Not Reported*.

**Table 4:** Summary of each neurofeedback protocol employed.

**Study**	**Imaging method**	**NF paradigm (participants)**	**Control paradigm (participants)**	**Number of sessions**	**Session duration (min.)**	**Session description**	**Feedback modality**	**Feedback description**	**Instructions**
**DEMENTIA**									
Surmeli et al. (2016)	EEG	participant-specific protocols (*N* = 20)	No	10–96 (avg. 45.0 ± 27.3)	60	NR	NR	NR	NR
Luijmes et al. (2016)	EEG	participant-specific protocols (*N* = 10)	No	30	30	4 blocks, with 5 min. breaks	Visual and auditory	Movie with varying contrast and beeping sound	No
Hohenfeld et al. (2017)	fMRI	↑ left parahipp. gyrus (*N* = 10)	CG1: ↑ left parahipp. gyrus (*N* = 16) CG2: ↑ left primary somatosensory cortex (*N* = 4)	3	60	4 blocks containing 12 trials (6 activation + 6 resting-state) of 40 s each	Visual	Thermometer bar	To remember footpath and/or count backwards
Hohenfeld et al. (2020)^*^	fMRI	↑ left parahipp. gyrus (*N* = 9)	↑ left parahipp. gyrus (*N* = 12)	3	60	4 blocks containing 12 trials (6 activation + 6 resting-state) of 40 s each	Visual	Thermometer bar	To remember footpath and/or count backwards
**MCI**									
Mendoza Laiz et al. (2018)^**^	EEG	↓ alpha (11–13 Hz) and ↑ beta (17–22 Hz) in C3, Cz, and C4 (*N* = 22 and 10)	No	5	60	60 trials with five different difficulty levels	Visual	Open or close virtual doors, or moving cursor	To imagine hand movements
Jang et al. (2019)	EEG	↑ beta (12–15 Hz) in F6 (*N* = 5)	No	16	45	9 trials of 5 min each	Visual	Moving a boat, or changing blurred flowers	To develop personal strategies
Jirayucharoensak et al. (2019)	EEG	↑ beta (12–32 Hz)/alpha (8–12 Hz) ratio in AF3 and AF4 (*N* = 58)	game (*N* = 38), CAU (*N* = 25)	20	30	5 blocks of 4–5 min separated by breaks of 2 min	Visual	Real-time game (5 different games)	Existent, but not reported
Lavy et al. (2019)	EEG	↑ alpha (8–10 Hz) in Pz (*N* = 11)	No	10	32	10 trials of 3 min each, separated by breaks of 10 s	Visual and auditory	Balls moving in 3D and beeping sound	To develop personal strategies
Li et al. (2020)	EEG	self-regulation of alpha (8–13 Hz) band and beta (13–30 Hz)/alpha (8–13 Hz) ratio (*N* = 40)	No	10	No limit	NR	Visual	NR	NR
Marlats et al. (2020)	EEG	↑ SMR (12–15 Hz) and ↓ theta (4–8 Hz) and beta (21–30 Hz) in Cz (*N* = 22)	No	20	75	NR	Visual and auditory	Animated graphics	Existent, but not reported

* *Methodological information detailed in previous publication from Hohenfeld et al. (2017)*.

** *Methodological information detailed in previous publication from Gomez-Pilar et al. (2016)*.

*CG, Control Group; NR, Not Reported; SMR, Sensory-Motor Rhythm; ↑, expected up-regulation; ↓, expected down-regulation*.

**Table 5:** Summary of outcomes from standardized cognitive assessment scales.

**Study**	**Within groups**	**Between groups**	**Follow-up**
**DEMENTIA**			
Surmeli et al. (2016)	NF: ↑ MMSE (19.00%)	N/A	N/A
Luijmes et al. (2016)	NF: ↑ CAMCOG (2.00%)	N/A	N/A
Hohenfeld et al. (2017)	NF: ↓ MoCA (1.00%) CG1: ↑ MoCA (3.97%) CG2: ↑ MoCA (0.83%)	NR	N/A
Hohenfeld et al. (2020)	NR	CG>NF: ↑ MoCA	N/A
**MCI**			
Mendoza Laiz et al. (2018)	NR	NR	N/A
Jang et al. (2019)	NF: ↑ MoCA-K (20.67%)	N/A	N/A
Jirayucharoensak et al. (2019)	NR	NR	N/A
Lavy et al. (2019)	NR	N/A	NR
Li et al. (2020)	NR	N/A	N/A
Marlats et al. (2020)	NF: ↑ MMSE (1.67%) ↑ MoCA (6.33%)	N/A	Regression of MoCA improvement

*CAMCOG, Cambridge Cognitive Examination; CG, Control Group; MMSE, Mini Mental Status Examination; MoCA, Montreal Cognitive Assessment; MoCA-K, Montreal Cognitive Assessment-Korean version; N/A, Not Apply; NF, Neurofeedback; NR, Not Reported; ↑, increased; ↓, decreased*.

**Table 6:** Summary of cognitive, behavioral, and neural outcomes.

**Study**	**Significant cognitive and behavioral changes**		**Significant neural changes**		**Follow-up**
	**Within groups**	**Between groups**	**Within groups**	**Between groups**
**DEMENTIA**					
Surmeli et al. (2016)	NF: ↑ orientation and recall MMSE subscales ↑ commission errors and reaction time variability TOVA subscales ↓ CGI	N/A	NF: ↓ in theta activity ↓ interhemispheric coherence	N/A	N/A
Luijmes et al. (2016)	No significant changes	N/A	NR	N/A	N/A
Hohenfeld et al. (2017)	NF: ↑ delayed recall of the visuospatial memory task of the VVM CG1: ↑ immediate recall condition visuospatial task of the VVM ↑ backward digit-span task of the WMS CG2: No significant differences	NR	No significant changes	NF>CG1: gray matter volume loss in the left parahipp. gyrus	N/A
Hohenfeld et al. (2020)	NR	Mixed model: Time effect for MoCA, VVM visuo-spatial memory test, and delayed recall Group differences for MoCA	NR	CG>NF: Activation in voxel clusters during task	N/A
**MCI**					
Mendoza Laiz et al. (2018)	NF1: ↑ visual perception ↑ spatial orientation ↑ receptive and expressive speech ↑ logical and immediate memory ↑ picture recognition and concepts NF2: ↑ picture recognition and concepts	No significant changes	NR	NR	N/A
Jang et al. (2019)	NF: ↑ CNSVS for composite and visual memory, cognitive flexibility, complex attention, reaction time, and executive function ↑ WM performance	N/A	NF: ↑ beta frequency	N/A	N/A
Jirayucharoensak et al. (2019)	NF: ↓ SWM_BER ↓ SWM_STR ↑ RVP_A' CG1 (game): SSP_SPAN	time × treatment groups on SWM_BER, SWM_STR, RVP_A′, and SSP_SPAN aMCI>HC: SWM_BER, SWM_STR, DMS_PER HC>aMCI: PRM_COR, DMS_COR	NR	NR	N/A
Lavy et al. (2019)	NR	N/A	NF: + corr. between peak alpha and session number ↑ composite memory following training ↑ non-verbal and verbal recall task	N/A	Sustained: composite memory improvement
Li et al. (2020)	NR	N/A	NF: ↑ connectivity in delta, theta, alpha and beta bands	N/A	N/A
Marlats et al. (2020)	NF: ↑ delayed recall of the RAVLT ↑ Forward digit span ↑ Mac Nair score ↓ GAS ↑ WAIS-IV	N/A	NF: ↑ overall theta and alpha power	N/A	Sustained: changes in theta and alpha power, and cognitive items Reduced: Forward digit span

*CAMCOG, Cambridge Cognitive Examination; CG, Control Group; CGI, Clinical Global Impression; CNSVS, Central Nervous System Vital Signs; DMS_COR, Delayed Matching to Sample Total Correct; DMS_PER, Delayed Matching to Sample Percent Correct; GAS, Goldberg Anxiety Scale; MMSE, Mini Mental Status Examination; N/A, Not Apply; NF, Neurofeedback; NR, Not Reported; PRM_COR, Pattern Recognition Memory Number Correct; RAVLT, Rey auditory verbal learning test; RVP_A', Rapid Visual Information Processing A prime; SSP_SPAN, Spatial Span Length; SWM_BER, Spatial Working Memory Between Error; SWM_STR, Spatial Working Memory Strategy; TOVA, Test of Variables of Attention; VVM, Visual and Verbal Memory Test; WAIS-IV, Wechsler Adult Intelligence Score-IV; WM, Working Memory task; WMS, Wechsler Memory Scale; ↑, increased; ↓, decreased*.

### 3.1. Overview of Studies

In 2016, two non-controlled experiments targeted the effect of EEG-based neurofeedback in patients with different types of dementia. Surmeli et al. (2016) conducted neurofeedback training with nine AD patients and eleven VD patients (mean age of 68.9 ± 10.6 years). All patients were receiving pharmacological treatment including cholinesterase inhibitors and antidepressant medication. The authors used individual, participant-specific training protocols that were based on the self-regulation of different EEG frequency bands across the scalp and with varying numbers of training sessions. After 10–96 training sessions, patients showed on average an improvement of 19.00% in their Mini Mental State Examination (MMSE) scores compared to baseline measures. Statistically significant changes were found in the orientation and recall sub-scales. This improvement is larger than what has been considered a meaningful clinical change (Howard et al., 2011; Andrews et al., 2019). However, the lack of control groups renders the interpretation difficult because the extent to which these changes are non-specific remains unclear (Ros et al., 2019; Sorger et al., 2019). Further, a reduction in the Clinical Global Impression (CGI) scale was also observed between the beginning and end of treatment. Moreover, the authors reported that after training 19 of 20 patients were withdrawn from their medication to treat dementia symptoms because their clinical improvement was considered sufficient.

Luijmes et al. (2016) evaluated patients with AD following the NINCDS-ADRDA guidelines (McKhann et al., 1984) (*N* = 10, 71.5 ± 6.8 years) who were receiving pharmacological treatment during 30 sessions of neurofeedback training. Using participant-specific designs, patients were instructed to self-regulate different EEG frequency bands on midline electrodes. A slight improvement (2.00%) in the Cambridge Cognitive Examination (CAMCOG) scale (primary outcome) was observed, with the highest improvement being reported in the Memory Learning sub-scale.

Hohenfeld et al. (2017) trained one group of prodromal AD patients (*N* = 10, 66.2 ± 8.9 years) and an age-matched healthy control group (CG1, *N* = 16, 63.5 ± 6.7 years) to up-regulate the fMRI signal of the left parahippocampal gyrus (PHG) in a non-randomized controlled study. A second age-matched healthy control group (CG2, *N* = 4, 64.8 ± 9.5 years) received sham feedback from the left primary somatosensory cortex. Participants were instructed to recall a footpath previously encoded in a visuospatial memory task, or to count backwards during rest periods. After three training sessions, the neurofeedback group with healthy participants showed significant improvements on the Montreal Cognitive Assessment test (MoCA, 3.97%) and increased flow of input functional connectivity in the left PHG. However, no significant changes in the MoCA scores were observed in the prodromal AD (−1.00%) or the sham control group (0.83%). In a secondary analysis, the authors compared clinical improvements and brain activations in different ROIs between sub-samples of prodromodal AD and healthy participants (Hohenfeld et al., 2020). The authors reported that the healthy control group showed significantly higher MoCA scores and activation of the left PHG following neurofeedback training.

Six other studies evaluated the effect of EEG-based neurofeedback training in patients with MCI. Mendoza Laiz et al. (2018) trained in a non-controlled study two groups of MCI patients (divided according to age ranges) with an EEG-based neurofeedback protocol to either down-regulate alpha (11–13 Hz) and or up-regulate beta (17–22 Hz) frequency bands in C3, Cz, and C4 electrodes using motor imagery of hand movements. The neurofeedback training comprised five sessions, which alternated with five working memory training sessions during which feedback was not provided. The working memory training task consisted of exercises related to different shapes, colors, and expressions. At the primary endpoint, the group including patients between 61 and 69 years of age (*N* = 22, 65.9 ± 2.2 years) showed significant improvement in several subscales of the Luria-DNA neuropsychological battery, including visual perception and orientation, receptive and expressive speech, and logical and immediate memory. Significant results were also observed for the group including patients between 70 and 81 years of age (*N* = 10, 73.1 ± 3.6), but only for the concept and picture recognition subscales. However, with the used study design it is not possible to disentangle the reason for this improvement as it may result from neurofeedback specific, neurofeedback non-specific effects (e.g., increased motivation or attention) that occurred in context of the motor imagery NF training, or from the working memory task.

Jang et al. (2019) published a non-controlled pilot EEG-neurofeedback study in which five MCI patients (66.6 ± 3.5 years) were instructed to develop their own strategies to up-regulate the beta (12–15 Hz) frequency band on the F6 location (allegedly recording the activity of the dorsolateral prefrontal cortex). After 16 training sessions, patients showed a significant improvement in the Korean version of the MoCA scale of 20.67%, an improvement that is larger than what is considered a minimum detectable change (Feeney et al., 2016). Moreover, patients showed improvements in several domains of the Central Nervous System Vital Signs (CNSVS) neurocognitive test battery, including the visual and composite memory, cognitive flexibility, complex attention, reaction time, and executive function. The authors also report a significantly better performance in an N-back working memory task at the primary outcome, and a significant correlation between the beta power and the session numbers. However, this single-group study did not control for non-specific, rendering the interpretation of the reported symptomatic changes difficult.

That same year, Lavy et al. (2019) conducted another non-controlled study with 11 patients suffering from MCI (70.0 ± 10.0 years). They trained patients to increase the EEG power in the alpha (8–10 Hz) frequency band over the central parietal region (Pz location) during 10 sessions. A positive correlation between the peak of alpha frequency and the session number was observed. After the intervention, patients showed increased performance on composite memory, verbal and non-verbal memory recall tasks. At the 4 weeks follow-up, the composite memory improvement, but not the improvements in other domains, was maintained. Again, the lack of a control group did not allow to control for unspecific effects, limiting the conclusiveness of these findings.

In 2020, Li et al. (2020) trained 40 MCI patients (54.3 ± 4.9 years) self-regulation of both power in the alpha (8–13 Hz) frequency band and the beta (13–30 Hz)/alpha (8–13 Hz) power ratio for 10 EEG-neurofeedback sessions. In a non-controlled setup, patients showed significant increase of the overall connectivity in delta, theta, alpha and beta bands. No behavioral outcome measures were reported.

Also in 2020, another non-controlled experiment reported by Marlats et al. (2020), aimed to entrain sensory-motor rhythm (SMR, 12–15 Hz) frequencies in twenty-two MCI patients (76.1 ± 5.9 years). Patients were trained to up-regulate SMR frequencies and down-regulate theta (4–8 Hz) and beta (21–30 Hz) frequency bands in the Cz electrode. The authors report that from the initial sample, only 20 participants completed the training. These patients presented improved scores in the MoCA scale (1.67%), as well as in the Goldberg Anxiety Scale (GAS) and the Wechsler Adult Intelligence Score IV (WAIS-IV). Significant increase in overall spectral power was also observed for theta and alpha bands. At the 4 weeks follow-up, cognitive and EEG changes were sustained, despite the MoCA scores having returned to baseline levels.

Finally, the only RCT (Jirayucharoensak et al., 2019) that was included in this review compared participants training to up-regulate the EEG beta (12–32 Hz)/alpha (8–12 Hz) ratio in AF3 and AF4 (*N* = 58, 71.7 ± 6.5 years) with two other groups: one control group (CG1, *N* = 36, 73.9 ± 6.2 years) engaged in a game-based physical exercise program (also referred as “exergame”), while the other control group received only care as usual (no more details reported, CG2, *N* = 25, 70.9 ± 5.1 years). However, samples in all three groups contained both healthy participants and MCI patients (please refer to Table 2). A significant treatment effect was reported for the experimental group in three subscales of the Cambridge Neuropsychological Test Automated Battery (CANTAB): spatial working memory between error, spatial working memory strategy, and rapid visual information processing. However, although the authors report higher effects in MCI patients vs. healthy participants, between group comparisons may be influenced by the heterogeneity within the experimental and groups. Because both groups contained healthy elderly participants as well as MCI patients, reported group differences may have been driven by CANTAB score improvements of (some) healthy elderly participants [note that in contrast to measurements, such as MMSE or MoCA, the CANTAB scale is a more complex test battery that prevents ceiling effects (Coull et al., 1995)].

### 3.2. Comparison of Cognitive Efficacy, Feasibility, and Safety Across Studies

**Figure 2:**
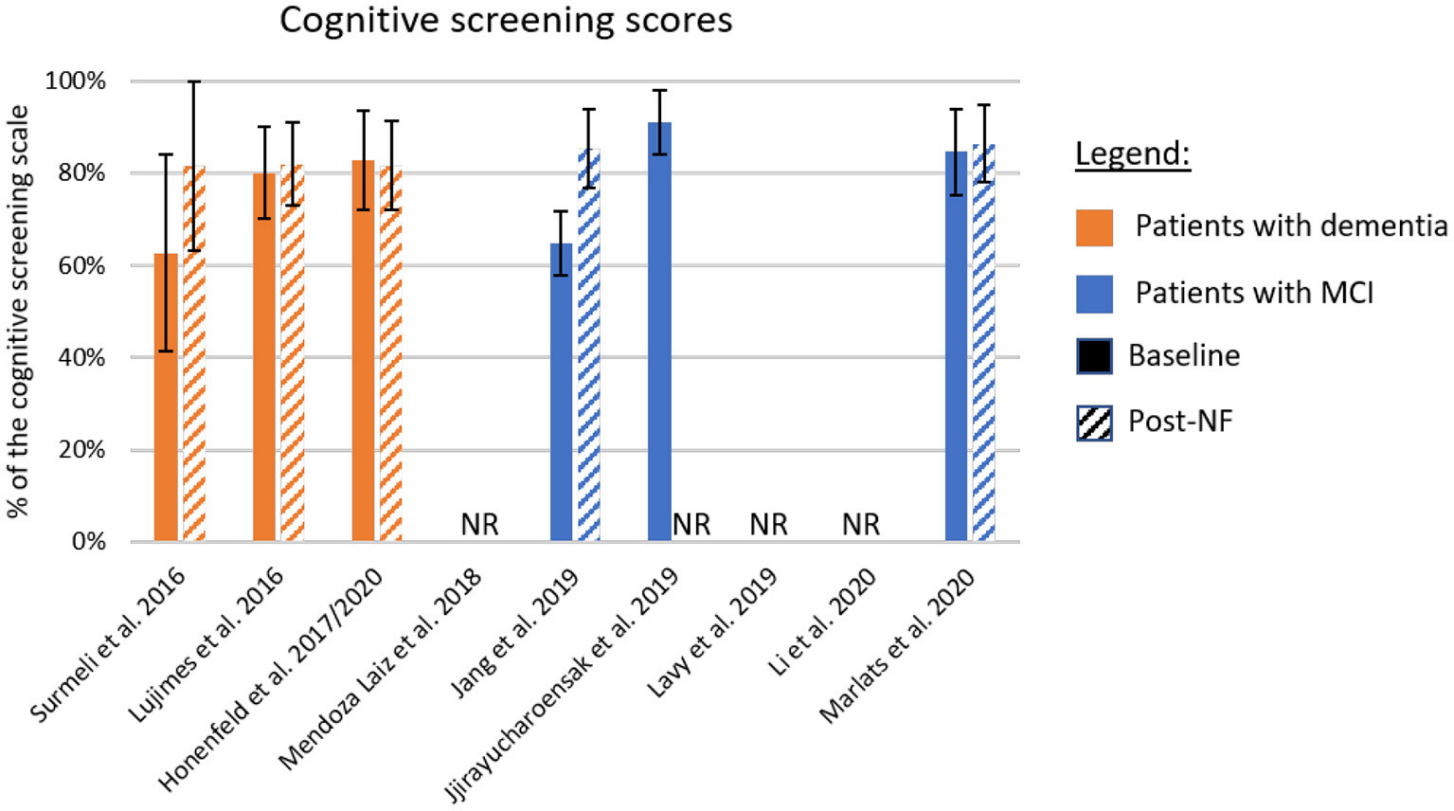
Summary of cognitive improvement according to standardized cognitive screening scales. The baseline and post-neurofeedback measures normalized as a percentage of the respective scales. In orange are the studies using patients with formal diagnosis of dementia, and in blue patients with mild-cognitive impairment (MCI). If the study did not report a primary outcome, we adopted a conservative approach and included in this chart results from the scale showing lower improvement. Solid bars represent the baseline scores, and dashed lines the post-intervention values. NF, Neurofeedback; NR, Not Reported.

The different cognitive screening instruments used for the diagnosis of dementia are highly correlated (Stewart et al., 2012; Trzepacz et al., 2015). Thus, we compared the clinical efficacy across studies after converting the different assessment scales to percentage values based on the scale maximum scores (Table 5 and Figure 2). The scores used in this comparison were the ones listed as primary outcome of each study (highlighted in bold in Table 2). For papers not identifying the primary outcome, we adopted a conservative approach and evaluated the scale with lower difference between baseline and the primary endpoint.

Figure 2 shows the baseline scores (solid bars) and the scores at the primary endpoint (dashed bars). Samples including patients with dementia are shown in orange, and those studying patients with MCI are shown in blue. One interesting factor is the low score presented by the MCI population in one study (Jang et al., 2019), suggesting that these participants may be already in transitory stages to dementia (Flicker et al., 1991). On the other hand, the study presenting higher scores at baseline (Jirayucharoensak et al., 2019) included both MCI patients and healthy participants in the neurofeedback group, which may explain the bar level.

Although many studies report improved performance in different memory tasks, or subscales, after intervention (Surmeli et al., 2016; Hohenfeld et al., 2017; Jang et al., 2019; Jirayucharoensak et al., 2019; Lavy et al., 2019), only five studies reported changes in standardized cognitive screening instruments (Figure 2). The averaged difference across these studies weighted by the sample size of each sample is 8.1%. However, although in two of these cases, differences were around 20.0% (Surmeli et al., 2016; Jang et al., 2019), in the three other studies these differences ranged from −1.0% (Hohenfeld et al., 2017) to 2.0% (Luijmes et al., 2016). Also, only one of the two studies that included follow-up sessions reported scores for a standardized cognitive screening scale (Marlats et al., 2020). In this study, the MoCA scores at follow-up returned to similar levels as observed at baseline, suggesting that clinical improvements might not be sustained long-term.

One possible explanation for such difference in behavioral/cognitive changes across studies might be the heterogeneity of training protocols. For instance, two studies employed participant-specific designs, with one study reporting substantial cognitive improvements (Surmeli et al., 2016) and one study reporting marginal cognitive improvements in patients (Luijmes et al., 2016). Moreover, all other EEG-based protocols included different channels or frequency ranges in their protocols (Mendoza Laiz et al., 2018; Jang et al., 2019; Jirayucharoensak et al., 2019; Lavy et al., 2019; Li et al., 2020; Marlats et al., 2020), limiting comparisons across studies. Future studies may benefit from translating (standardized) protocols that have been successfully tested in elderly participants (Laborda-Sánchez and Cansino, 2021) to evaluate their potential efficacy in patients suffering from dementia or cognitive impairment. However, we note that a previous review of this field identified mostly non-RCT studies Laborda-Sánchez and Cansino (2021), limiting conclusions that can be drawn about the effectiveness of these protocols. Alternatively, protocols that were previously tested in healthy participants could be translated to patients. For instance, a recent meta-analysis (Yeh et al., 2020) evaluated RCTs of EEG neurofeedback training of the alpha frequency band in healthy individuals. Findings suggested that this protocol may be an effective option to improve working memory and episodic memory. Specifically, the authors found moderate effect size for both memory categories, whilst further analysis showed little risk for publication bias among these RCTs (Yeh et al., 2020). Efficacy in improving cognitive function has thus far mainly been demonstrated in young and healthy individuals. As a next step, it will require translation of this protocol to patients suffering from dementia or MCI. Further, here we identified only one protocol that used fMRI neurofeedback training (Hohenfeld et al., 2017). Hence, there remains substantial scope for future neurofeedback studies to explore the potential of fMRI targets (e.g., subcortical areas, such as the hippocampus) to treat core dementia and MCI symptoms (e.g., memory loss) (Ruan et al., 2016).

Another relevant aspect is the lack of control groups and conditions in many experimental designs. In this review, we only identified one RCT (Jirayucharoensak et al., 2019) and one controlled protocol (Hohenfeld et al., 2017, 2020), which stands in stark contrast to the 16 RCTs that were recently reported in a meta-analysis of EEG protocols for memory improvement in healthy participants (Yeh et al., 2020). Another difference worth noticing is that most trials that were included in the meta-analysis by Yeh et al. featured larger sample sizes per group, and several trials included multiple arms, enabling them to control for different non-specific effects (Sorger et al., 2019). For the only RCT included in our review, the authors compared neurofeedback training with treatment as usual, including both MCI patients and healthy participants in all groups (Jirayucharoensak et al., 2019). As described above, such group composition may have influenced within and between group comparisons. Regarding the other controlled study included in this review, the control group (which received sham feedback) was composed exclusively by healthy participants (Hohenfeld et al., 2017), which limits any clinical conclusions. Taken together, the lack of adequately controlled studies for neurofeedback training targeting cognitive symptoms in dementia and MCI patients severely limits interpretations.

On a positive note, neurofeedback protocols are rarely associated with side effects (Hawkinson et al., 2012). Potential side effects experienced by patients include potential physical discomfort experienced before [e.g., during EEG cap preparation and calibration (Nijholt et al., 2011)] or during training sessions [e.g., claustrophobia due to the physical restriction in fMRI scanners (Sulzer et al., 2013)]. Noteworthy, only one study reported withdraws before the primary endpoint (Marlats et al., 2020), one other study reported drop-offs before the follow-up completion (Lavy et al., 2019), and three studies reported data exclusion due to technical problems or excessive noise in the recordings (Hohenfeld et al., 2017, 2020; Jang et al., 2019). However, none of these studies reported serious side effects for the neurofeedback intervention. These findings are in line with those reported by systematic reviews of other clinical and non-clinical neurofeedback applications (Kohl et al., 2020; Tursic et al., 2020; Trambaiolli et al., 2021). The safety and feasibility of such experimental setups is especially important in dementia research (as discussed in section 4), since changes in environment, interaction with experimenters, and task demands can trigger emotional and psychological distress in elderly participants (Hellström et al., 2007; Novek and Wilkinson, 2019).

### 3.3. Experimental Design and Reporting Quality

**Figure 3:**
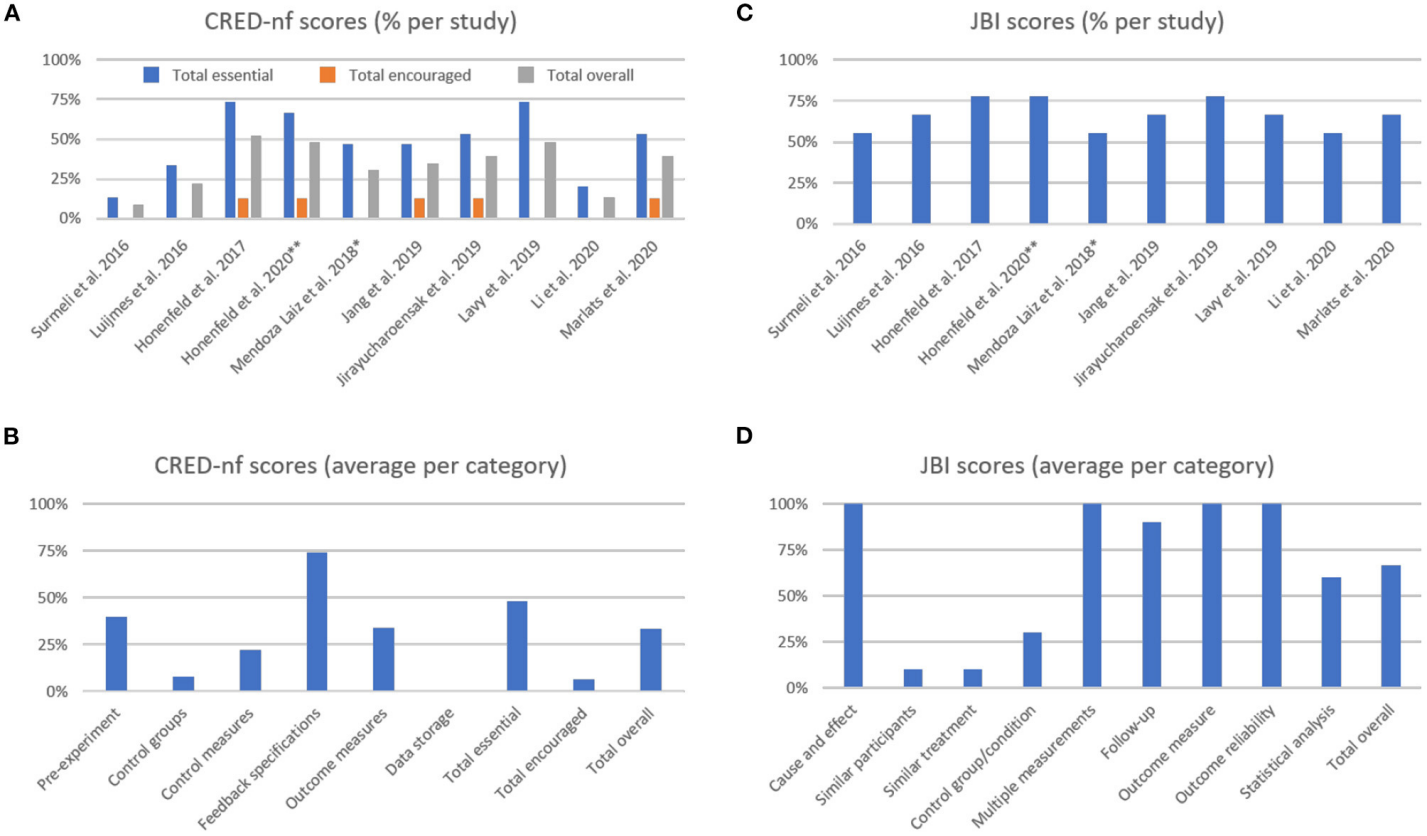
The Consensus on the Reporting and Experimental Design of Neurofeedback studies (CRED-nf) percentage scores **(A)** per study, and **(B)** averaged per category. The Joanna Briggs Institute (JBI) averaged percentage scores **(C)** per study, and **(D)** averaged per category. **Methodological information detailed in previous publication from Hohenfeld et al. (2017); *Methodological information detailed in previous publication from Gomez-Pilar et al. (2016).

Following our second aim, we present a systematic evaluation of studies' experimental design and reporting quality using the CRED-nf checklist (Ros et al., 2019) and the JBI critical appraisal tools (Tufanaru et al., 2017) (Figure 3, please see Supplementary Material for detailed scoring for each study).

The average CRED-nf score (as percentage) across studies was 50.8 ± 20.8% for essential, 6.3 ± 6.7% for encouraged, and 35.3 ± 14.8% for all items. As shown in Figure 3A, only one study had an overall score (gray bars) above 50.0%, while two studies showed total scores that were below 25.0%. In Figure 3B, it is notable that the items with the lowest scores are related to control groups and conditions, and data sharing (the latter was not fulfilled by any study included in this review). Comparing the essential, encouraged, and total CRED-nf ratings for studies in dementia with other fields, these scores are substantially lower than those reported in systematic reviews about EEG and fMRI neurofeedback training in depression (~65.0, 13.0, and 47.0%, respectively, Trambaiolli et al., 2021), and fNIRS neurofeedback in non-clinical/clinical populations (63.0, 10.0, and 45.0%, respectively, Kohl et al., 2020).

For the JBI checklist the averaged score (as percentage) across studies was 68.1 ± 9.3% (Figure 3C), with the lowest scores being related to the similarities between groups, and control design (Figure 3D). Comparing the JBI ratings for studies in dementia patients with other fields, we see a similarity to those reported in systematic reviews about EEG and fMRI neurofeedback training in depression (mean 68.89%, Trambaiolli et al., 2021), fMRI neurofeedback in stroke patients (mean 70.00%, Wang et al., 2018), and fNIRS neurofeedback in non-clinical/clinical populations (mean 61.67%, Kohl et al., 2020). However, an important difference is that the number of included studies in these previous reviews (24, 33, and 22, in the reviews on depression, stroke, and fNIRS, respectively) is higher than the number of studies included in the current review (*N* = 10).

The highest scores in the CRED-nf checklist were obtained in the “Feedback specification” category. Items from this group check for the instrumental component of the feedback protocol (Ros et al., 2019). These relatively good scores are in agreement with other applications of neurofeedback (Kohl et al., 2020; Trambaiolli et al., 2021), although specific details about applied real-time artifact correction procedures are often still lacking for most neurofeedback studies (Heunis et al., 2020).

On the other hand, some of the lowest scores are related to control groups and conditions. This effect is strongly related to the low scores in the “Similar participants,” “Similar treatment,” and “Control group/conditions” from the JBI checklist. This finding can be explained, for example, by the fact that 7 out of 10 studies in this review used single-group designs (Luijmes et al., 2016; Surmeli et al., 2016; Mendoza Laiz et al., 2018; Jang et al., 2019; Lavy et al., 2019; Li et al., 2020; Marlats et al., 2020). This approach is relevant in early stages of a new intervention, for instance to assess safety as well as technical and clinical feasibility (similar to Phase I Clinical Trial designs) (Sorger et al., 2019). However, given that several unspecific effects contribute to the overall outcome of neurofeedback training (Micoulaud-Franchi and Fovet, 2018; Ros et al., 2019), appropriate controlled experiments are much needed to validate clinical applications (Thibault et al., 2018; Sorger et al., 2019).

Finally, low scores in “Pre-experiment” and “Data storage” items indicate that study registration and data sharing, i.e., transparent research practices, are still lacking. A similar conclusion was drawn in our recent systematic reviews of fNIRS neurofeedback training (Kohl et al., 2020) as well as for neurofeedback training in depression (Trambaiolli et al., 2021).

## 4. Future Directions

Studies evaluating potential clinical interventions for dementia require careful planning and preparation because they face several methodological, analytical and ethical challenges (Hellström et al., 2007; Richard et al., 2012; Ritchie et al., 2015; Novek and Wilkinson, 2019). For instance, patients may present with a mix of amyloid, vascular or other pathologies associated with dementia (Schneider et al., 2007). Thus, traditional single intervention RCTs may not be the most adequate approach for dementia (Richard et al., 2012). Another challenge concerns when assessments should be performed (Ritchie et al., 2015), since follow-up studies are commonly affected by high mortality indices (Agüero-Torres et al., 1999; Andersen et al., 2010). Further, participation in complex interventions, such as neurofeedback training may result in psychological side-effects. For instance, tasks and interactions with researchers may cause distress (Hellström et al., 2007; Novek and Wilkinson, 2019), or even trigger aggressive behaviors in patients (Whall et al., 1997; Orengo et al., 2008). With this in mind, we understand that research using neurofeedback protocols in this population should be carefully designed to minimize risks and optimize potential benefits. Thus, in line with the final aim of this review, we provide recommendations for future research evaluating the effects of neurofeedback training in patients suffering from dementia or MCI.

### 4.1. Comprehensive Clinical Documentation

Although standardized cognitive screening scales were used as part of diagnostic process, four out of ten studies did not report baseline scores according to these scales. Further aspects of a comprehensive clinical documentation include a detailed description of previous pharmacological treatments and other possible comorbidities. To ensure reliable clinical results and to allow comparison between studies, future studies should report baseline comparisons for formal and standardized assessment scores (e.g., MMSE, MoCA, or CAMCOG). Regarding the choice of the primary and secondary outcome measures, we recommend using cognitive testing tools that allow capturing the core cognitive processes that are being targeted (Lubianiker et al., 2019). Further, because dementia affects also other psychological domains, such as mood, we encourage researchers to use adequate scales that allow monitoring potential mood changes. For instance, lower mood and elevated anxiety are often observed in patients suffering from dementia and may impact disease trajectories (Paterniti et al., 2002). Previous work has demonstrated substantial therapeutic effects of neurofeedback training to treat mood or anxiety (Tolin et al., 2020; Trambaiolli et al., 2021), partly based on protocols that employed memory based self-regulation strategies (Young et al., 2017) or entrained areas of the hippocampal formation (see active control group in Mehler et al., 2018). Noteworthy, mood and anxiety influence cognitive performance, and may hence mediate observed effects (McDermott and Ebmeier, 2009; de Vito et al., 2019). Standardized clinical mood scales may further be complemented by measurements that allow disentangling changes in symptoms that occur within and between training sessions (Mehler et al., 2021). In addition to clinical scales, which bear the risk of a reductionist view of treatment effects, using additional qualitative or semi-quantitative measures (e.g., testimonials of patients, relatives, and care-givers) may be worthwhile. To ensure a comprehensive clinical documentation of patients, we recommend measuring and reporting well-established molecular biomarkers and risk factors for dementia. Noteworthy, only one (Hohenfeld et al., 2017) of the included protocols reported such data. In this context we want to highlight the recent research from Skouras et al. who found an association between a clinical biomarker for AD and self-regulation success (please note that the study was not included in our systematic review because the authors did not report cognitive or clinical outcome measurements). First, the authors showed that participants carrying APOE-ε4 alleles showed lower self-regulation performance during hippocampal down-regulation compared to non-carriers (Skouras et al., 2019). Second, they reported reduced eigenvector centrality (i.e., less influence based on iterative whole-brain connectomics) in the anterior cingulate cortex and primary motor cortex during hippocampus down-regulation when comparing cognitively unimpaired participants who had abnormal levels of CSF amyloid-β peptide 42 with cognitively unimpaired participants with lower CSF amyloid-β peptide 42 levels (Skouras et al., 2020). This example shows how biomarker data can provide valuable insights about the neurofeedback literacy in patients with dementia. For instance, carriers of neurobiological biomarkers may need more training sessions to achieve a comparable performance as non-carriers, and the training protocol may need to be designed accordingly. Lastly, information about recruitment and attainment should be documented alongside with other phases within the study (from screening to follow-up) using a CONSORT diagram (Moher et al., 2012).

### 4.2. Appropriately Controlled Study Designs

One challenge when evaluating neurofeedback training is to control adequately for unspecific effects, e.g., reported cognitive improvement of patients may be partly or even mostly due to motivational factors, personal positive believes, and engaging in the experiment (Thibault and Raz, 2017; Thibault et al., 2017). Several studies have reported cognitive improvements that were larger than minimally detectable changes (MDC), reliable change indices (RCI) or even minimum clinically important differences (MCID), suggesting that changes may be both reliable and clinically meaningful. However, it requires properly controlled study designs to determine if such effects are specific to the used neurofeedback protocol. In this context, an experimental framework for proper design of control groups or conditions in neurofeedback experiments was recently described by Sorger et al. (2019). For studies evaluating patients with dementia or MCI, different control conditions should be considered. To control for unspecific effects, future studies should consider control conditions which would emulate the same experimental environment and reward process (Sorger et al., 2019). Possible strategies include the presentation of sham feedback (Hohenfeld et al., 2017), targeting different areas or networks, or more recent approaches, such as the “randomized ROI” condition, where participants of the control group are randomly assigned to different subsets of neural control targets (Lubianiker et al., 2019). Further, studies need to evaluate the participant's remained blind to their assigned group. These aspects are relevant as control belief can directly affect the training performance and future engagement (Witte et al., 2013). Since one main goal of neurofeedback treatment studies in dementia is to evaluate possible clinical benefits and current treatment options are limited, the evaluation of neurofeedback alongside standard-of-care interventions is in particular desirable (Cox et al., 2016). Lastly, in two controlled studies identified in this review, the control groups were completely (Hohenfeld et al., 2017) or partially (Jirayucharoensak et al., 2019) composed of healthy elderly participants. Comparisons between different study populations may invalidate conclusions regarding possible therapeutic effects. Thus, studies should include groups with similar clinical characteristics, e.g., they should be matched for diagnosis, age, gender, and education level.

### 4.3. Appropriately Powered Study Designs

Notably, many studies reviewed here neither reported sampling plans, nor were they labeled accordingly as a “pilot,” or “proof-of-concept.” Hence, the robustness of clinical findings remain limited (Ros et al., 2019; Sorger et al., 2019). Future studies should be powered appropriately to allow detecting relevant effects and provide more precise estimates. Sampling plans should be based on plausible effect sizes, i.e., the smallest relevant effect sizes one can afford to miss at a given type-I and II error rates (Algermissen and Mehler, 2018), rather than estimates from small pilot studies. The latter tend to overestimate true effects, and when used to inform power calculations, may bias follow-up studies, increasing the risk for type-I errors (Albers and Lakens, 2018). Ideally, the choice of the smallest relevant is informed by meta-research, e.g., clinically relevant effect sizes reported for the chosen primary outcome measure (Howard et al., 2011; Kopecek et al., 2017; Andrews et al., 2019). We acknowledge, however, that achieving sufficient recruitment may be challenging in the targeted populations (e.g., due to inclusion or exclusion criteria) and that researchers may face considerable attrition rates (e.g., in particular for longitudinal designs). Hence, we recommend researchers to explore recent statistical developments. For instance, repeated measurements with mixed-effects modeling may increase statistical power (Aarts et al., 2015). Further, potential recruitment difficulties could be mitigated by the use of flexible statistical approaches, such as sequential Bayesian sampling (Schönbrodt and Wagenmakers, 2018), which allows to sample data until a pre-defined evidence threshold (often expressed as Bayes Factor) is reached. Sequential Bayesian sampling provides higher statistical sensitivity compared to fixed-N sampling plans, in particular for small effects (Schönbrodt and Wagenmakers, 2018). Sampling plans could be either based on effects from a neural outcome measure, such as self-regulation success (e.g., see Mehler et al., 2020) or a behavioral/clinical outcome measure. A detailed description of the sampling plan should ideally be preregistered (see also section 4.9). Lastly, we recommend for studies that fail to reject the null hypothesis should conduct follow-up tests that allow to establish whether reported outcomes are conclusive (Mehler et al., 2019).

### 4.4. Specific Demands for Studies With Elderly Cohorts

As mentioned, protocols focusing on patients with dementia and MCI require specific methodological considerations (Hellström et al., 2007; Richard et al., 2012; Ritchie et al., 2015; Novek and Wilkinson, 2019). For instance, large-scale RCTs may benefit from multiple acquisition sites to achieve the recruitment goals, which will demand data collection and preprocessing methods to reduce the effects of scanner/amplifier variability (Teipel et al., 2017). Also, extensive and challenging sessions may cause discomfort and distress, possibly triggering aggressive behaviors in patients (Whall et al., 1997; Orengo et al., 2008). Thus, the protocol should include shorter experimental sessions to improve tolerability, but specific pipelines will be necessary to compensate for shorter data length, movement-related noise, among other effects (Harms et al., 2018). Finally, the inclusion and exclusion criteria should consider potential comorbidities and possible consequences of pharmacological treatments in the neural signal of interest (Evangelisti et al., 2019).

### 4.5. Inclusion of Transfer Sessions and Follow-Up Evaluation

Similar to other cognitive training approaches, the mechanisms of transferring the learned cognitive strategies to real-life situations should be evaluated (Greenwood and Parasuraman, 2016). For example, healthy participants were able to transfer strategies learned during neurofeedback training to situations without feedback presentation [e.g., during self-regulation of somatomotor cortices (Auer et al., 2015)]. Further, participants could successfully use these cognitive strategies, for instance, motor (Auer et al., 2015) or visual imagery (Robineau et al., 2017), even months after the end of the experiment. Additionally, effects in behavior and symptoms should also be monitored during transfer sessions and longitudinally after neurofeedback training. For instance, longitudinal therapeutic effects can be found for the benefits of neurofeedback training in psychiatric populations (Mehler et al., 2018; Rance et al., 2018). However, none of the studies reviewed here included transfer sessions and only two studies reported follow-up evaluation. Although both studies that reported follow-up assessments found sustained memory benefits, effects in general cognitive assessment scales (Marlats et al., 2020) or neural signatures (Lavy et al., 2019) returned to baseline levels. Thus, in addition to transfer sessions, long-term monitoring should be included in future study designs.

### 4.6. Standardization of Protocols

The main goal of neurofeedback studies that are tailored toward patients suffering from dementia is to achieve cognitive and clinical improvements. Ideally, these are accompanied by (neuroplastic) changes in neural outcomes of, for instance, the targeted brain region (e.g., a percent signal change in the fMRI signal) or electrical frequency (e.g., changes in power of the EEG signal). Moreover, functional or structural changes on network level should be explored. Although participant-specific designs (Luijmes et al., 2016; Surmeli et al., 2016) present value due to individual variability, comparability between studies could be further enhanced once feature extraction methods and feedback presentation methods are standardized. Further, standardized definitions on measures, such as self-regulation training success are needed to explore dose-response relationships. Recent consensus statements have suggested options for standardizing methodological approaches (Paret et al., 2019) and reporting measures of self-regulation performance (Ros et al., 2019). Lastly, with regards to the clinical effectiveness of neurofeedback training protocols for AD/MCI, definitions for treatment responders and non-responders should be used (e.g., MDCs, RCIs, or MCIDs) (Howard et al., 2011; Kopecek et al., 2017; Andrews et al., 2019) to allow comparing its effectiveness to other interventions or clinical neurofeedback applications in other conditions.

### 4.7. Investigation of New Neurofeedback Targets

Dementia and MCI are complex diseases, with the neural substrates expanding beyond local activity (Ruan et al., 2016; Chandra et al., 2019). Moreover, disorders considered risk factors for dementia, such as depression, seem to show mechanistic overlapping (Kim and Kim, 2021). In this sense, further exploration of new neurofeedback targets focusing on functional networks is much needed. For instance, protocols using fMRI can focus on recent methods of neurofeedback targeting functional connectivity or network patterns (Rana et al., 2016). In protocols using EEG, the use of source-level functional connectivity might be an option. In this context, we would like to highlight the case reports from Koberda (2014) (not included in our sample given the exclusion criteria listed in Table 1). In this study, patients trained with LORETA-based neurofeedback from different brain regions presented reorganization of EEG-based functional connectivity. These results suggest that LORETA-based functional connectivity may also be an option for future protocols targeting functional networks.

### 4.8. Comprehensive Reporting

As previously mentioned, some studies in our sample did not provide sufficient detail about sample characteristics, the methodological setup, or the relationship between the outcomes and the learned control of the neurofeedback protocol. We encourage researchers to use the CRED-nf (Ros et al., 2019), that was used in this review to evaluate published studies. We note that this checklist was recently published and, consequently unavailable to the authors of most studies included in this review. However, these checklists provide a standardized orientation for design and reporting practices. These can be used for the planning phase of future experiments (see also the CRED-nf online application rtfin.org/CREDnf), including early phase pilot and proof-of-concept studies, and thereby help the field progressing toward higher quality RCTs that will facilitate achieving more conclusive findings.

### 4.9. Open Science Practices

Neurofeedback training in dementia is a relatively new field. Thus, in order to accelerate the development of this novel clinical application, as well as to increase transparency and reliability of proposed protocols, we strongly recommend that researchers pre-register their protocols comprehensively to provide transparency about a priori hypotheses and delineate planned from exploratory hypotheses (see for a detailed example, Mehler et al., 2020). We further encourage authors to share data and code that support their results. In particular fMRI neurofeedback researchers can take advantage of substantial progress in the neuroimaging field when it comes to standardized pipelines, e.g., when using the pipeline proposed by Nichols et al. (2017), which incorporates best practices to promote open data in functional neuroimaging. Overall, we recommend to explore potential benefits of open science research practices while considering possible challenges (Allen and Mehler, 2019), e.g., when translating a complex paradigm from healthy participants to patients.

### 4.10. Test of Mobile Approaches

One common complication for patients suffering from dementia are impaired mobility (Härlein et al., 2009) and functional dependencies (Livingston et al., 2017). These symptoms may hinder participants from participating in studies at research sides. The investigation of mobile protocols may provide an attractive alternative to address these challenges and may hence be worthwhile to investigate in these populations. For instance, low-cost and portable EEG equipment using dry electrodes have shown to achieve similar results to state-of-the-art wired laboratory EEG systems in event-related paradigms (De Vos et al., 2014; Ries et al., 2014; Cassani et al., 2017) with the addition of extra pre-processing data enhancement steps. Other possible options include the use of wearable fNIRS systems that allow mobile use outside of laboratory settings (Kohl et al., 2020), e.g., to conduct experiments or intervention studies in naturalistic environments (Balardin et al., 2017). In this context, recent advances in the development of mobile/modular EEG-fNIRS (von Lühmann et al., 2016) and mobile/unshielded MEG systems (Zhang et al., 2020) may be considered in future protocols. Moreover, mobile neurofeedback systems for home use could be integrated in mobile-health approaches, such as cognitive training applications to promote more autonomous aging for elderly patients with beginning cognitive impairment (Cisotto et al., 2021). Altogether, mobile applications can facilitate study participation and will allow for scalable employment of neurofeedback interventions.

### 4.11. Use of Hybrid Systems

Hybrid systems combine different types of signals in one brain-computer interface (BCI) or neurofeedback system (Pfurtscheller et al., 2010). For example, they may combine signals from different neuroimaging modalities (e.g., electric and hemodynamic responses), neural patterns from the same neuroimaging modality (e.g., SMRs and evoked potentials measured with EEG), or from a neuroimaging modality and a non-neural source (e.g., EEG signals and heart-rate variability) (for a complete overview, please refer to Banville and Falk, 2016). Using hybrid systems may help increasing the robustness of the system and facilitate classifying the user's mental processes (Fazli et al., 2012). Specifically, it may allow monitoring levels of stress, attention and mental workload, and then optimize feedback and task demands individually to the user, enabling more “neuroergonomic” and effective designs of BCI or neurofeedback interventions (Albuquerque et al., 2020; Parent et al., 2020). Hence, hybrid systems seem in particular attractive to test for populations, such as patients suffering from dementia.

## 5. Conclusion

Neurofeedback presents a potential non-invasive intervention to slow down or even reverse cognitive decline in patients suffering from dementia or mild cognitive impairment. Our review of the current literature suggests that while patients have shown significant improvements in memory tasks or some subscales of standardized cognitive tests, clinical efficacy still remains undetermined. The design and reporting quality of studies published to date largely lag behind current best research practices with regards to their design and reporting quality. Some main issues include the lack of (1) control conditions, (2) sampling plans, (3) randomized treatment allocation, (4) rater blinding, and (5) use of standardized cognitive screening instruments. These issues render the evaluation of clinical effects difficult and require improvements in future studies. We therefore close this review with a set of recommendations, including more comprehensive clinical documentation, adequate control conditions, follow-up investigations, reporting quality, and use of transparent research practices. We further encourage exploring the potential for outside-the-lab neurofeedback applications with portable devices.

## Author Contributions

LT conceptualized the review with input from all other authors, selected the studies, extracted the data, assessed the quality of the studies, drafted, and revised the manuscript. RC extracted the data, assessed the quality of the studies, and revised the manuscript. DM and TF supervised the drafting of the manuscript and revised it. All authors contributed to the article and approved the submitted version.

## Conflict of Interest

The authors declare that the research was conducted in the absence of any commercial or financial relationships that could be construed as a potential conflict of interest.
