# Supplementary material for: Neurofeedback and the Aging Brain: A Systematic Review of Training Protocols for Dementia and Mild Cognitive Impairment
Source: Front Aging Neurosci. 2021 Jun 9;13:682683. doi: 10.3389/fnagi.2021.682683 (PMC8221422; doi:10.3389/fnagi.2021.682683)
Supplement: Supplementary file 1 [file Presentation_1.pdf]

Supplementary Material

1 DETAILS ABOUT CRED-NF RATINGS

We used the recently published "Consensus on the Reporting and Experimental Design of clinical and cognitive-behavioural NeuroFeedback studies" (CRED-nf) checklist Ros et al. (2019) to evaluate the experimental design and reporting quality of the studies included in our review. However, most of the studies we evaluated were conducted before the existence of the checklist, we adapted some of the original CRED-nf items and applied more lenient criteria. We used similar criteria as in previous neurofeedback reviews Kohl et al. (2020); Trambaiolli et al. (2021). Table S1 shows the detailed results of the ratings per study.

Item 1b: Justify sample size

- If no power analysis was conducted, but the study was clearly labeled as *pilot*, *proof-of concept/proof-of-principle*, *exploratory*, or *feasibility study*, we rated this item as "Yes".
- If a study used a database from a previous paper, the score in this item was the same as the previous study.

Item 2a: Employ control group(s) or control condition(s)

- If the control group included subjects with dementia or mild cogntivie impairment, we rated this item as "Yes".
- If the control group only included healthy subjects, we rated this item as "No".

Item 2b: When leveraging experimental designs where a double-blind is possible, use a double-blind

- If the study was single-blind but discussed this choice (justifying and proposing solutions for future experiments), we rated this item as "Yes".

Item 3a: Collect data on psychosocial factors

- If the study included data related to motivational/expectation scores, self-evaluation measurements, or standardized questionnaires reporting at least one measure describing efficacy, competence, motivation, among others, we rated this item as "Yes".
- If a study used a database from a previous paper, the score in this item was the same as the previous study.

Item 3b: Report whether participants were provided with a strategy

- If the paper reported at least a brief description of strategies provided, we rated this item as "Yes".

- If a study used a database, or the experimental paradigm from a previous paper, the score in this item was the same as the original study.

Item 3c: Report the strategies participants used

- If the paper reported at least a qualitative list of strategies used by the participants, we rated this item as “Yes”.
- If the study mention specific instructions, but did not assess and report the strategies used, we rate this item as “No”.
- If a study used a database, or the experimental paradigm from a previous paper, the score in this item was the same as the original study.

Item 3d: Report methods used for online data processing and artifact correction

- If a study used a database, or the experimental paradigm from a previous paper, the score in this item was the same as the original study.

Item 3e: Report condition and group effects for artifacts

- If a study used a database, or the experimental paradigm from a previous paper, the score in this item was the same as the original study.

Item 4a: Report how the online-feature extraction was defined

- If a study used a database, or the experimental paradigm from a previous paper, the score in this item was the same as the original study.

Item 4b: Report and justify the reinforcement schedule

- If a study used a database, or the experimental paradigm from a previous paper, the score in this item was the same as the original study.

Item 4c: Report the feedback modality and content

- If the study reported at least a feedback threshold, or a description of the feedback schedule (e.g., continuous feedback, intermittent feedback, or every few seconds), we rated this item as “Yes”.
- If a study used a database, or the experimental paradigm from a previous paper, the score in this item was the same as the original study.

Item 4d: Collect and report all brain activity variable(s) and/or contrasts used for feedback, as displayed to experimental participants

- If a study reported at least the contrast for regulation vs. rest condition for the region(s) of interest (ROIs) or channels, used for feedback, we rated this item as "Yes". Reporting or plotting main effects for the conditions rest and regulation only was not required.
- If a study used a database, or the experimental paradigm from a previous paper, the score in this item was the same as the original study.

Item 4e: Report the hardware and software used

- If a study used a database, or the experimental paradigm from a previous paper, the score in this item was the same as the original study.

Item 5b: Plot within-session and between-session regulation blocks of feedback variable(s), as well as pre-to-post resting baselines or contrasts

- If the study plotted time courses on at least one of the two temporal levels (within- or between-session), we rated this item as "Yes".
- If a study used a database from a previous paper, the score in this item was the same as the previous study.

Item 6a: Include measures of clinical or behavioural significance, defined a priori, and describe whether they were reached

- If the study reported the number of subjects that changed the clinical range according to predefined scales, we rated this item as "Yes".
- If the study reported the clinical scores per patient using standardized scales (in a way that allows further evaluation of clinical significance), we rated this item as "Yes".

Item 6b: Run correlational analyses between regulation success and behavioural outcomes

- If the study evaluated correlations including at least one measure from neural data, we rated this item as "Yes".
- If the study evaluated correlations including only behavioral, or clinical variables, we rated this item as "No".

Table S1. Detailed scores for the CRED-nf checklist. 1 = reported; 0 = not reported; * = details described in Hohenfeld et al. (2017); ** = details described in Gomez-Pilar et al. (2016).

	CRED-nf	Status	Surmeli et al. (2016)	Luijmes et al. (2016)	Hohenfeld et al. (2017)	Hohenfeld et al. (2020)	Mendoza Laiz et al. (2018)	Jang et al. (2019)	Jirayucharoensak et al. (2019)	Lavy et al. (2019)	Li et al. (2020)	Marlats et al. (2020)
1a	Pre-registration	encouraged	N	N	N	N	N	Y	N	N	N	Y
1b	Sample planning	essential	N	Y	Y	Y	N	Y	N	Y	N	Y
2a	Control group(s) or condition(s)	essential	N	N	Y	Y	N	N	Y	N	N	N
2b	Blinding	essential	N	N	N	N	N	N	N	N	N	N
2c	Blind rater	encouraged	N	N	N	N	N	N	N	N	N	N
2d	Blinding assessed	encouraged	N	N	N	N	N	N	N	N	N	N
2e	Standard-of-care intervention	encouraged	N	N	N	N	N	N	Y	N	N	N
3a	Psychosocial factors assessment	encouraged	N	N	Y	Y*	N	N	N	N	N	N
3b	Report strategies provided	essential	N	N	Y	Y*	Y**	N	N	Y	N	Y
3c	Report strategies used	encouraged	N	N	N	N*	N	N	N	N	N	N
3d	Online-data processing	essential	N	N	N	N*	Y**	N	Y	Y	N	Y
3e	Group effects for artifacts	encouraged	N	N	N	N*	N	N	N	N	N	N
4a	Online-feature extraction	essential	N	N	Y	Y*	Y**	Y	Y	Y	N	Y
4b	Reinforcement schedule	essential	N	Y	Y	Y*	Y**	N	Y	Y	N	Y
4c	Feedback	essential	N	Y	Y	Y*	Y**	Y	Y	Y	Y	Y
4d	Variable(s) used for feedback	essential	N	N	Y	Y*	N	Y	N	Y	N	N
4e	Hardware and software	essential	Y	Y	Y	Y*	Y	Y	Y	Y	Y	Y
5a	Neurofeedback regulation success	essential	N	N	N	N	N	N	N	Y	N	N
5b	Plot of feedback variable(s)	essential	N	N	Y	N	N	Y	N	Y	N	N
5c	Group comparison	essential	N	N	Y	Y	N	N	Y	N	N	N
6a	Clinical or behavioral significance	essential	Y	Y	Y	Y	Y	Y	Y	Y	Y	Y
6b	Correlation between outcomes	essential	N	N	N	N	N	N	N	N	N	N
7a	Open-access data storage	encouraged	N	N	N	N	N	N	N	N	N	N

2 DETAILS ABOUT THE JBI SCORES

When using the checklist for quasi-experimental studies of the Joanna Briggs Institute (JBI) critical appraisal tools Tufanaru et al. (2017), we had to adapt some of the items to fit the neurofeedback field. We used similar criteria as in previous neurofeedback reviews Kohl et al. (2020); Trambaiolli et al. (2021). Table S2 shows the detailed results of the ratings per study.

Item 2: Similar participants in compared groups

- If the baseline measures or demographics were similar (even if a study did not check for similarity), we rated this item as "Yes".

Item 3: Similar treatment in compared groups

- If the study reported both groups receiving similar treatments before the beginning of the neurofeedback experiment (for example, similar medications or psychotherapeutic interventions), we rated this item as "Yes".
- If the study did not report anything (presumption of innocence), we also rated this item as "Yes".

Item 4: Existence of control group/condition

- If the control group included patients with dementia or mild cognitive impairment, we rated this item as "Yes".
- If the study included only healthy patients in the control group, we rated this study as "No".

Item 5: Multiple measurement points of the outcome

- As discussed by Kohl et al. (2020), the neurofeedback field is relatively novel, and several studies included in this review are pilot or proof-of-concept studies. Thus, requiring four measurement points seemed too rigorous. If the studies presented two measurements in at least two separate days (for example, a classical pre-post design), we rated this item as "Yes".

Item 6: Completion of follow-up

- No separate follow-up measurements after training were required to fulfill this item. If the primary endpoint was completed and the study reported and managed the existence of the dropouts, we rated this item as "Yes".

- If a study did not have at least two separate sessions for pre-post assessment on two different days, we rated this item as “Not Applicable”.

Item 9: Appropriate statistical methods

- If no power analysis was conducted, but the study was clearly labeled as *pilot*, *proof-of concept/proof-of-principle*, *exploratory*, or *feasibility study*, we rated this item as “Yes”.
- If a study used a database from a previous paper, the score in this item was the same as the previous study.

Table S2. Detailed scores for the JBI checklist. Y = Yes; N = No; N/A = Not-Applicable.

JBI	Surmeli et al. (2016)	Luijmes et al. (2016)	Hohenfeld et al. (2017)	Hohenfeld et al. (2020)	Mendoza Laiz et al. (2018)	Jang et al. (2019)	Jirayucharoensak et al. (2019)	Lavy et al. (2019)	Li et al. (2020)	Marlats et al. (2020)
1. Cause and effect	Y	Y	Y	Y	Y	Y	Y	Y	Y	Y
2. Similar participants	N/A	N/A	N	N	N/A	N/A	Y	N/A	N/A	N/A
3. Similar treatment	N/A	N/A	N/A	N/A	N/A	N/A	Y	N/A	N/A	N/A
4. Control group/condition	N	N	Y	Y	N	N	Y	N	N/A	N/A
5. Multiple measurements (pre/post)	Y	Y	Y	Y	Y	Y	Y	Y	Y	Y
6. Follow up complete	Y	Y	Y	Y	Y	Y	N	Y	Y	Y
7. Outcome measure	Y	Y	Y	Y	Y	Y	Y	Y	Y	Y
8. Outcome reliability	Y	Y	Y	Y	Y	Y	Y	Y	Y	Y
9. Statistical analysis	N	Y	Y	Y	N	Y	N	Y	N	Y

REFERENCES

Gomez-Pilar, J., Corralejo, R., Nicolas-Alonso, L. F., Álvarez, D., and Hornero, R. (2016). Neurofeedback training with a motor imagery-based bci: neurocognitive improvements and eeg changes in the elderly. *Medical & biological engineering & computing* 54, 1655–1666

Hohenfeld, C., Kuhn, H., Müller, C., Nellessen, N., Ketteler, S., Heinecke, A., et al. (2020). Changes in brain activation related to visuo-spatial memory after real-time fmri neurofeedback training in healthy elderly and alzheimer's disease. *Behavioural Brain Research* 381, 112435

Hohenfeld, C., Nellessen, N., Dogan, I., Kuhn, H., Müller, C., Papa, F., et al. (2017). Cognitive improvement and brain changes after real-time functional mri neurofeedback training in healthy elderly and prodromal alzheimer's disease. *Frontiers in neurology* 8, 384

Jang, J.-H., Kim, J., Park, G., Kim, H., Jung, E.-S., Cha, J.-y., et al. (2019). Beta wave enhancement neurofeedback improves cognitive functions in patients with mild cognitive impairment: A preliminary pilot study. *Medicine* 98

Jirayucharoensak, S., Israsena, P., Pan-ngum, S., Hemrungrojn, S., and Maes, M. (2019). A game-based neurofeedback training system to enhance cognitive performance in healthy elderly subjects and in patients with amnestic mild cognitive impairment. *Clinical interventions in aging* 14, 347

Kohl, S. H., Mehler, D., Lührs, M., Thibault, R. T., Konrad, K., and Sorger, B. (2020). The potential of functional near-infrared spectroscopy-based neurofeedback—a systematic review and recommendations for best practice. *Frontiers in Neuroscience* 14, 594

Lavy, Y., Dwolatzky, T., Kaplan, Z., Guez, J., and Todder, D. (2019). Neurofeedback improves memory and peak alpha frequency in individuals with mild cognitive impairment. *Applied psychophysiology and biofeedback* 44, 41–49

Li, X., Zhang, J., Li, X.-D., Cui, W., and Su, R. (2020). Neurofeedback training for brain functional connectivity improvement in mild cognitive impairment. *JOURNAL OF MEDICAL AND BIOLOGICAL ENGINEERING*

Luijmes, R. E., Pouwels, S., and Boonman, J. (2016). The effectiveness of neurofeedback on cognitive functioning in patients with alzheimer's disease: Preliminary results. *Neurophysiologie Clinique/Clinical Neurophysiology* 46, 179–187

Marlats, F., Bao, G., Chevallier, S., Boubaya, M., Djabelkhir-Jemmi, L., Wu, Y.-H., et al. (2020). Smr/theta neurofeedback training improves cognitive performance and eeg activity in elderly with mild cognitive impairment: A pilot study. *Frontiers in Aging Neuroscience* 12

Mendoza Laiz, N., Del Valle Diaz, S., Rioja Collado, N., Gomez-Pilar, J., and Hornero, R. (2018). Potential benefits of a cognitive training program in mild cognitive impairment (mci). *Restorative neurology and neuroscience* 36, 207–213

Ros, T., Enriquez-Geppert, S., Zotev, V., Young, K. D., Wood, G., Whitfield-Gabrieli, S., et al. (2019). Consensus on the reporting and experimental design of clinical and cognitive-behavioural neurofeedback studies (cred-nf checklist). *Brain*

Surmeli, T., Eralp, E., Mustafazade, I., Kos, H., Özer, G. E., and Surmeli, O. H. (2016). Quantitative eeg neurometric analysis–guided neurofeedback treatment in dementia: 20 cases. how neurometric analysis is important for the treatment of dementia and as a biomarker? *Clinical EEG and neuroscience* 47, 118–133

Trambaiolli, L. R., Kohl, S. H., Linden, D. E., and Mehler, D. M. (2021). Neurofeedback training in major depressive disorder: a systematic review of clinical efficacy, study quality and reporting practices. *Neuroscience & Biobehavioral Reviews* 125, 33–56

Tufanaru, C., Munn, Z., Aromataris, E., Campbell, J., and Hopp, L. (2017). Chapter 3: Systematic reviews of effectiveness. *Joanna Briggs Institute Reviewer's Manual. The Joanna Briggs Institute*
